# Complete genome sequence of *Kribbella flavida* type strain (IFO 14399^T^)

**DOI:** 10.4056/sigs.731321

**Published:** 2010-03-30

**Authors:** Rüdiger Pukall, Alla Lapidus, Tijana Glavina Del Rio, Alex Copeland, Hope Tice, Jan-Fang Cheng, Susan Lucas, Feng Chen, Matt Nolan, Kurt LaButti, Amrita Pati, Natalia Ivanova, Konstantinos Mavromatis, Natalia Mikhailova, Sam Pitluck, David Bruce, Lynne Goodwin, Miriam Land, Loren Hauser, Yun-Juan Chang, Cynthia D. Jeffries, Amy Chen, Krishna Palaniappan, Patrick Chain, Manfred Rohde, Markus Göker, Jim Bristow, Jonathan A. Eisen, Victor Markowitz, Philip Hugenholtz, Nikos C. Kyrpides, Hans-Peter Klenk, Thomas Brettin

**Affiliations:** 1 DSMZ – German Collection of Microorganisms and Cell Cultures GmbH, Braunschweig, Germany; 2 DOE Joint Genome Institute, Walnut Creek, California, USA; 3 Los Alamos National Laboratory, Bioscience Division, Los Alamos, New Mexico, USA; 4 Biological Data Management and Technology Center, Lawrence Berkeley National Laboratory, Berkeley, California, USA; 5 Oak Ridge National Laboratory, Oak Ridge, Tennessee, USA; 6 HZI – Helmholtz Centre for Infection Research, Braunschweig, Germany; 7 University of California Davis Genome Center, Davis, California, USA

**Keywords:** *Actinobacteria*, aerobic, soil, mycelia, LL-diaminopimelic acid, *Propionibacterineae*, *Nocardioidaceae*, GEBA

## Abstract

The genus *Kribbella* consists of 15 species, with *Kribbella flavida* (Park *et al*. 1999) as the type species. The name *Kribbella* was formed from the acronym of the Korea Research Institute of Bioscience and Biotechnology, KRIBB. Strains of the various *Kribbella* species were originally isolated from soil, potato, alum slate mine, patinas of catacombs or from horse racecourses. Here we describe the features of *K. flavida* together with the complete genome sequence and annotation. In addition to the 5.3 Mbp genome of *Nocardioides* sp. JS614, this is only the second completed genome sequence of the family *Nocardioidaceae*. The 7,579,488 bp long genome with its 7,086 protein-coding and 60 RNA genes and is part of the *** G****enomic* *** E****ncyclopedia of* *** B****acteria and* *** A****rchaea * project.

## Introduction

Strain IFO 14399^T^ (= DSM 17836 = KCTC 9580 = JCM 10339 = NBRC 14399) is the type strain the species *Kribbella flavida*, which is the type species of the genus *Kribbella*. Strain IFO 14399^T^ was originally isolated from soil in China and first described as ‘*Nocardioides fulvus*’ by Ruan and Zhang, 1979 [1]. In 1999, the strain was reclassified into the novel genus *Kribbella* on the basis of comparative chemotaxonomic and 16S rRNA sequence analysis [2]. *K. flavida* exhibits mycelia on several media used for growing the strain. The mycelium consists of hyphae, which are extensively branched and penetrate into the agar medium. The hyphae often fragment into rod to coccus-like elements [2]. Here we present a summary classification and a set of features for *K. flavida* IFO 14399^T^, together with the description of the complete genomic sequencing and annotation.

## Classification and features

**Figure 1:**
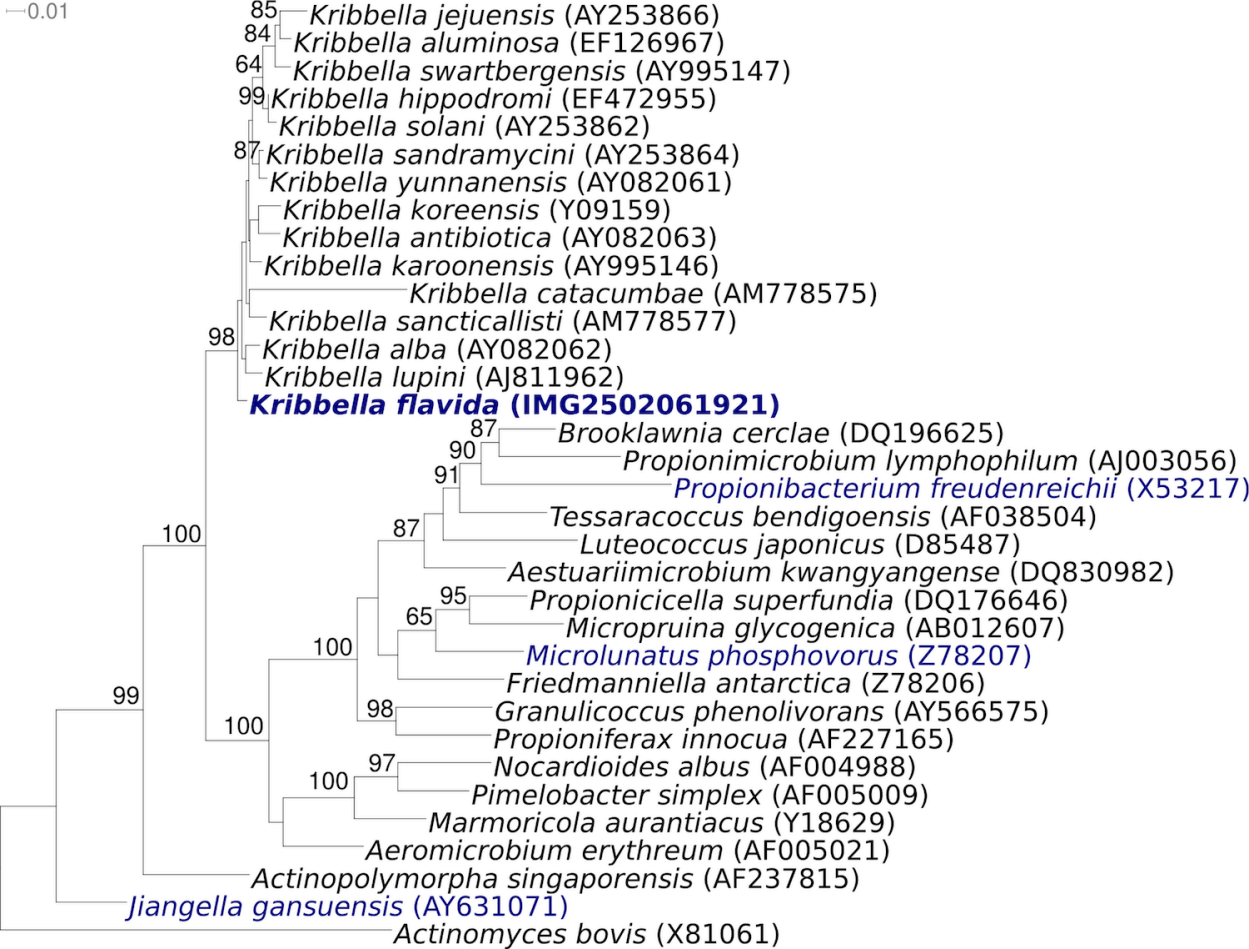
Tree highlighting the position of *K. flavida* IFO 14399^T^ relative to the other type strains of the genus *Kribbella* and the type strains of the other genera within the families *Nocardioidaceae* and *Propionibacteriaceae*. The tree was inferred from 1,343 aligned characters [3,4] of the 16S rRNA gene sequence under the maximum likelihood criterion [5] and rooted in accordance with current taxonomy. The branches are scaled in terms of the expected number of substitutions per site. Numbers above branches are support values from 1,000 bootstrap replicates if larger than 60%. Lineages with type strain genome sequencing projects registered in GOLD [6] are shown in blue, published genomes in bold.

The type strain IFO 14399^T^ was isolated from soil in China. Genbank contains only one additional 16S rRNA gene sequence with at least 99% similarity, derived from a strain isolated from scabby potatoes (EU80972). No phylotypes from environmental samples or genomic surveys be directly linked to *K. flavida*, indicating rare occurrence of the species in so far screened habitats (October 2009). Figure 1 shows the phylogenetic neighborhood of *K. flavida* IFO 14399^T^ in a 16S rRNA based tree. The sequence of the two 16S rRNA genes in the genome of strain 14399^T^ differ by two nucleotides from each other and by up to two nucleotides from the previously published 16S rRNA sequence generated from KACC 20258 (AY253863).

**Figure 2:**
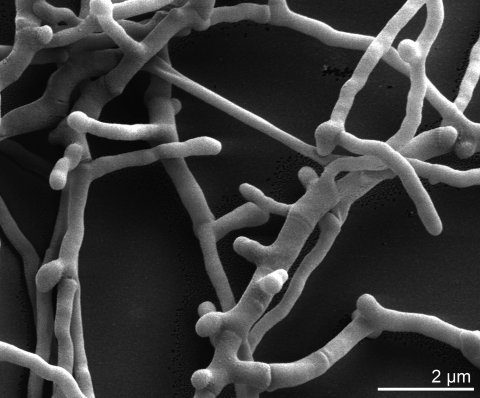
Scanning electron micrograph of *K. flavida* IFO 14399^T^

*K. flavida* is a Gram-positive, aerobic and non-acid-fast actinomycete (Table 1), characterized by primary mycelium (Figure 2), with branched hyphae that penetrate into the agar medium. Aerial mycelium is also developed and can break up into short to elongated rod-like elements. Growth occurs between pH 5 and 9 and between 20 and 37°C. The strain shows positive activity for catalase, oxidase and urease. It utilizes D-glucose, D-cellobiose, maltose, D-melibiose, sucrose, D-trehalose, melezitose, D-raffinose, adonitol, myo-inositol, D-mannitol, inulin, disodium succinate and disodium fumarate as sole carbon and energy source [2].

**Table 1:** Classification and general features of *K. flavida* IFO 14399^T^ according to the MIGS recommendations [7]

**MIGS ID**	**Property**	**Term**	**Evidence code**
	Current classification	Domain *Bacteria*	TAS [8]
		Phylum *Actinobacteria*	TAS [9]
		Class *Actinobacteria*	TAS [10]
		Order *Actinomycetales*	TAS [11]
		Suborder *Propionibacterineae*	TAS [10]
		Family *Nocardioidaceae*	TAS [12]
		Genus *Kribbella*	TAS [2]
		Species *Kribbella flavida*	TAS [2]
		Type strain IFO 14399	TAS [2]
	Gram stain	positive	TAS [2]
	Cell shape	hyphae, fragmented into rod to coccoid elements	TAS [2]
	Motility	nonmotile	NAS
	Sporulation	nonsporulating	NAS
	Temperature range	20°C-37°C	TAS [2]
	Optimum temperature	not reported	
	Salinity	not reported	
MIGS-22	Oxygen requirement	strictly aerobic	TAS [2]
	Carbon source	saccharolytic	TAS [2]
	Energy source	carbohydrates	TAS [2]
MIGS-6	Habitat	soil	TAS [2]
MIGS-15	Biotic relationship	free living	NAS
MIGS-14	Pathogenicity	none	NAS
	Biosafety level	1	TAS [13]
	Isolation	soil	TAS [1,2]
MIGS-4	Geographic location	Beijing, China	TAS [1]
MIGS-5	Sample collection time		NAS
MIGS-4.1 MIGS-4.2	Latitude Longitude	39.55 116.25	NAS
MIGS-4.3	Depth	not reported	
MIGS-4.4	Altitude	not reported	

Evidence codes - IDA: Inferred from Direct Assay (first time in publication); TAS: Traceable Author Statement (i.e., a direct report exists in the literature); NAS: Non-traceable Author Statement (i.e., not directly observed for the living, isolated sample, but based on a generally accepted property for the species, or anecdotal evidence). These evidence codes are from of the Gene Ontology project [14]. If the evidence code is IDA, then the property was directly observed by one of the authors or an expert mentioned in the acknowledgements.

### Chemotaxonomy

One of the meaningful characteristics of the genus *Kribbella* is the presence of LL-diaminopimelic acid as the diagnostic diamino acid in the cell wall peptidoglycan [2]. The predominant menaquinone is a tetrahydrogenated menaquinone with nine isoprenoid units MK-9(H_4_) [2]. The major fatty acids detected in *K. flavida* are anteiso-C_15:0_ and iso-C_16:0_ [2]. Phosphatidylcholine is the main polar lipid [2]. The genus *Kribbella* differs from other LL-diaminopimelic acid and MK-9(H4) containing taxa, by having a typical hyphal morphology [2].

## Genome sequencing and annotation

### Genome project history

This organism was selected for sequencing on the basis of its phylogenetic position, and is part of the *** G****enomic* *** E****ncyclopedia of* *** B****acteria and* *** A****rchaea * project. The genome project is deposited in the Genome OnLine Database [15] and the complete genome sequence is deposited in GenBank. Sequencing, finishing and annotation were performed by the DOE Joint Genome Institute (JGI). A summary of the project information is shown in Table 2.

**Table 2:** Genome sequencing project information

**MIGS ID**	**Property**	**Term**
MIGS-31	Finishing quality	Finished
MIGS-28	Libraries used	Two Sanger libraries: 8kb pMCL200 and fosmid pcc1Fos One 454 pyrosequence standard library and one Standard Illumina library
MIGS-29	Sequencing platforms	ABI3730, 454 GS FLX, Illumina GA
MIGS-31.2	Sequencing coverage	7.4× Sanger; 13.4× pyrosequence
MIGS-30	Assemblers	Newbler 1.1.01.20, phrap
MIGS-32	Gene calling method	Prodigal, GenePRIMP
	INSDC ID	CP001736
	Genbank Date of Release	January 13, 2010
	GOLD ID	Gc01192
	NCBI project ID	21089
	Database: IMG-GEBA	2501939632
MIGS-13	Source material identifier	DSM 17836
	Project relevance	Tree of Life, GEBA

### Growth conditions and DNA isolation

*K. flavida* IFO 14399^T^, DSM 17836, was grown in DSM medium 830 [15] at 28°C. DNA was isolated from 1-1.5 g of cell paste using Qiagen Genomic 500 DNA Kit (Qiagen, Hilden, Germany) following the manufacturer's instructions with modification st/FT for cell lysis according to Wu *et al*. [16].

### Genome sequencing and assembly

The genome was sequenced using a combination of Sanger and 454 sequencing platforms. All general aspects of library construction and sequencing can be found on the JGI website. 454 Pyrosequencing reads were assembled using the Newbler assembler version 1.1.01.20 (Roche). Large Newbler contigs were broken into 8,548 overlapping fragments of 1,000 bp and entered into assembly as pseudo-reads. The sequences were assigned quality scores based on Newbler consensus q-scores with modifications to account for overlap redundancy and to adjust inflated q-scores. A hybrid 454/Sanger assembly was made using the parallel phrap assembler (High Performance Software, LLC). Possible mis-assemblies were corrected with Dupfinisher [17] or transposon bombing of bridging clones (Epicentre Biotechnologies, Madison, WI). Gaps between contigs were closed by editing in Consed, custom primer walk or PCR amplification. A total of 2,850 Sanger finishing reads were produced to close gaps, to resolve repetitive regions, and to raise the quality of the finished sequence. Illumina reads were used to improve the final consensus quality using an in-house developed tool (the Polisher). The error rate of the completed genome sequence is less than 1 in 100,000. Together all sequence types provided 51.2× coverage of the genome. The final assembly contains 59,008 Sanger and 433,053 pyrosequence reads.

### Genome annotation

Genes were identified using Prodigal [18] as part of the Oak Ridge National Laboratory genome annotation pipeline, followed by a round of manual curation using the JGI GenePRIMP pipeline [19]. The predicted CDSs were translated and used to search the National Center for Biotechnology Information (NCBI) nonredundant database, UniProt, TIGRFam, Pfam, PRIAM, KEGG, COG, and InterPro databases. Additional gene prediction analysis and manual functional annotation was performed within the Integrated Microbial Genomes Expert Review (IMG-ER) platform [20].

## Genome properties

**Figure 3:**
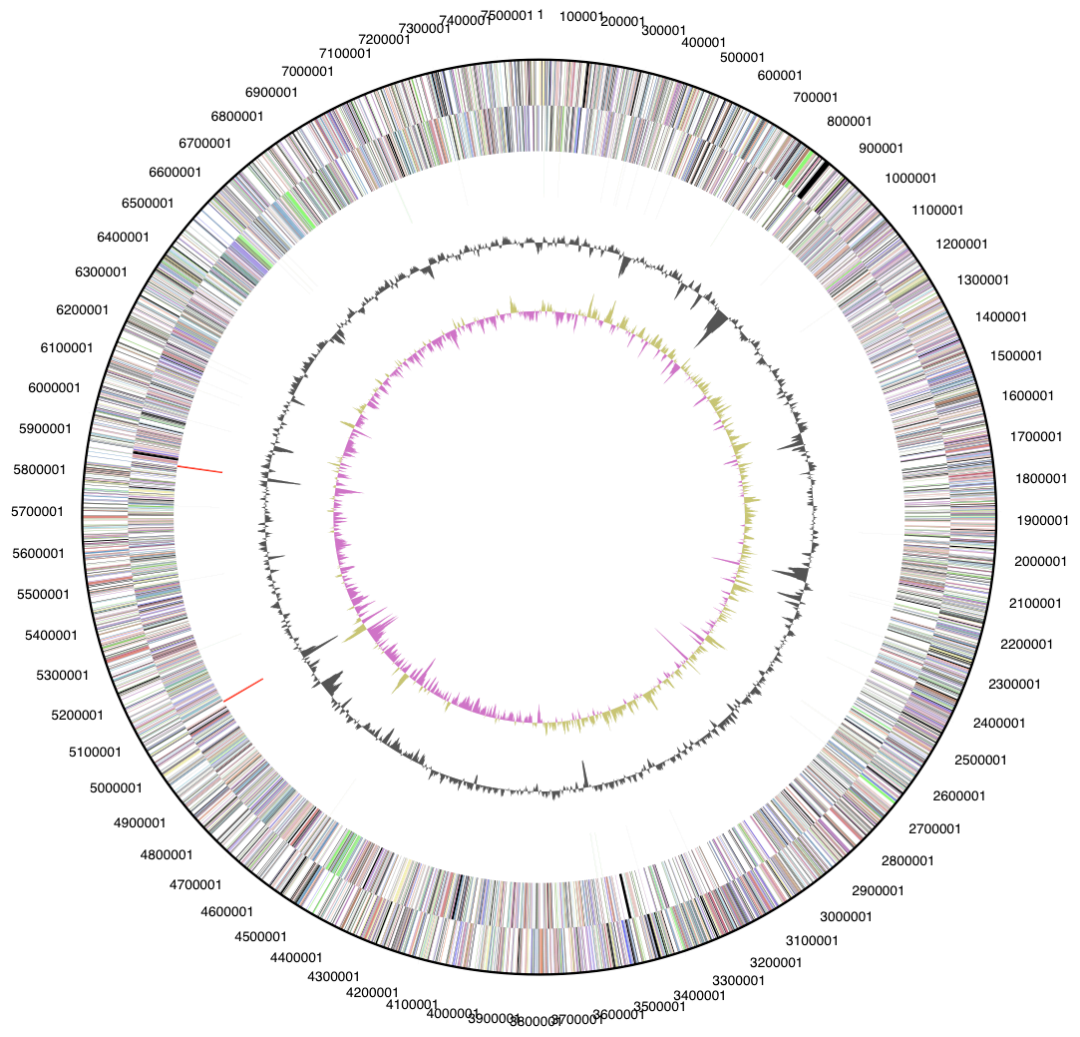
Graphical circular map of the chromosome. From outside to the center: Genes on forward strand (color by COG categories), Genes on reverse strand (color by COG categories), RNA genes (tRNAs green, rRNAs red, other RNAs black), GC content, GC skew.

The genome is 7,579,488 bp long with a 70.6% GC content (Table 3 and Figure 3). Of the 7,146 genes predicted, 7,086 were protein-coding genes, and 60 RNAs; 143 pseudogenes were also identified. The majority of the protein-coding genes (70.7%) were assigned with a putative function while those remaining were annotated as hypothetical proteins. The distribution of genes into COGs functional categories is summarized in Table 4.

**Table 3:** Genome Statistics

**Attribute**	**Value**	**% of Total**
Genome size (bp)	7,579,488	100.00%
DNA coding region (bp)	6,893,122	90.94%
DNA G+C content (bp)	5,348,686	70.57%
Number of replicons	1	
Extrachromosomal elements	0	
Total genes	7,146	100.00%
RNA genes	60	0.84%
rRNA operons	2	
Protein-coding genes	7,086	99.16%
Pseudo genes	143	2.00%
Genes with function prediction	5,049	70.65%
Genes in paralog clusters	1,595	22.32%
Genes assigned to COGs	4,877	68.25%
Genes assigned Pfam domains	5,174	72.40%
Genes with signal peptides	1,721	24.08%
Genes with transmembrane helices	1,675	23.44%
CRISPR repeats	0	0

**Table 4:** Number of genes associated with the general COG functional categories

**Code**	**Value**	**%age**	**Description**
J	225	4.1	Translation, ribosomal structure and biogenesis
A	2	0.0	RNA processing and modification
K	762	13.8	Transcription
L	184	3.3	Replication, recombination and repair
B	1	0.0	Chromatin structure and dynamics
D	38	0.7	Cell cycle control, cell division, chromosome partitioning
Y	0	0.0	Nuclear structure
V	136	2.5	Defense mechanisms
T	261	4.7	Signal transduction mechanisms
M	239	4.3	Cell wall/membrane biogenesis
N	2	0.0	Cell motility
Z	2	0.0	Cytoskeleton
W	0	0.0	Extracellular structures
U	46	0.8	Intracellular trafficking and secretion
O	143	2.6	Posttranslational modification, protein turnover, chaperones
C	308	5.6	Energy production and conversion
G	636	11.5	Carbohydrate transport and metabolism
E	397	7.2	Amino acid transport and metabolism
F	100	1.9	Nucleotide transport and metabolism
H	264	4.8	Coenzyme transport and metabolism
I	212	3.8	Lipid transport and metabolism
P	218	3.9	Inorganic ion transport and metabolism
Q	175	3.2	Secondary metabolites biosynthesis, transport and catabolism
R	802	14.5	General function prediction only
S	367	6.7	Function unknown
-	2,269	31.8	Not in COGs

## References

[1] Ruan J Zhang Y Two new species of *Nocardioides*. Acta Microbiol Sin 1979; 19:347-352

[2] Park YH Yoon JH Shin YK Suzuki K Kudo T Seino A Kim HJ Lee JS Lee ST Classification of '*Nocardioides fulvus*' IFO 14399 and *Nocardioides* sp. ATCC 39419 in *Kribbella* gen. nov., as *Kribbella flavida* sp. nov. and *Kribbella sandramycini* sp. nov. Int J Syst Bacteriol 1999; 49:743-752 10.1099/00207713-49-2-743 10319498

[3] Castresana J Selection of conserved blocks from multiple alignments for their use in phylogenetic analysis. Mol Biol Evol 2000; 17:540-552 10742046 10.1093/oxfordjournals.molbev.a026334

[4] Lee C Grasso C Sharlow MF Multiple sequence alignment using partial order graphs. Bioinformatics 2002; 18:452-464 10.1093/bioinformatics/18.3.452 11934745

[5] Stamatakis A Hoover P Rougemont J A Rapid Bootstrap Algorithm for the RAxML Web Servers. Syst Biol 2008; 57:758-771 10.1080/10635150802429642 18853362

[6] Liolios K Chen IM Mavromatis K Tavernarakis N Hugenholtz P Markowitz VM Kyrpides NC The Genomes On Line Database (GOLD) in 2009: status of genomic and metagenomic projects and their associated metadata. Nucleic Acids Res 2010; 38:D346-D354 10.1093/nar/gkp848 19914934 PMC2808860

[7] Field D Garrity G Gray T Morrison N Selengut J Sterk P Tatusova T Thomson N Allen MJ Angiuoli SV The minimum information about a genome sequence (MIGS) specification. Nat Biotechnol 2008; 26:541-547 10.1038/nbt1360 18464787 PMC2409278

[8] Woese CR Kandler O Wheelis ML Towards a natural system of organisms: proposal for the domains *Archaea*, *Bacteria*, and *Eucarya*. Proc Natl Acad Sci USA 1990; 87:4576-4579 10.1073/pnas.87.12.4576 2112744 PMC54159

[9] Garrity GM, Holt JG. The Road Map to the Manual. *In*: Garrity GM, Boone DR, Castenholz RW (eds), *Bergey’s Manual of Systematic Bacteriology*, Second Edition, Springer, New York, 2001, p. 119-169.

[10] Stackebrandt E Rainey FA Ward-Rainey NL Proposal for a new hierarchic classification system, *Actinobacteria* classis nov. Int J Syst Bacteriol 1997; 47:479-491 10.1099/00207713-47-2-479

[11] Skerman VBD McGowan V Sneath PHA Approved Lists of Bacterial Names. Int J Syst Bacteriol 1980; 30:225-420 10.1099/00207713-30-1-225 20806452

[12] Nesterenko OA Kvasnikov EI Nogina TM *Nocardioidaceae* fam. nov., a new family of the order *Actinomycetales* Buchanan 1917. Mikrobiol Zh 1985; 47:3-12

[13] Classification of *Bacteria* and *Archaea* in risk groups www.baua.de TRBA 466.

[14] Ashburner M Ball CA Blake JA Botstein D Butler H Cherry JM Davis AP Dolinski K Dwight SS Eppig JT Gene Ontology: tool for the unification of biology. Nat Genet 2000; 25:25-29 10.1038/75556 10802651 PMC3037419

[15] List of growth media used at DSMZ: http://www.dsmz.de/microorganisms/ media_list.php

[16] Wu D Hugenholtz P Mavromatis K Pukall R Dalin E Ivanova N Kunin V Goodwin L Wu M Tindall BJ A phylogeny-driven genomic encyclopedia of *Bacteria* and *Archaea*. Nature 2009; 462:1056-1060 10.1038/nature08656 20033048 PMC3073058

[17] Sims D Brettin T Detter JC Han C Lapidus A Copeland A Glavina Del Rio T Nolan M Chen F Lucas S Complete genome of *Kytococcus sedentarius* type strain (541^T^). Stand Genomic Sci 2009; 1:12-20 10.4056/sigs.761 PMC3035214 21304632

[18] Hyatt D Chen GL Locascio PF Land ML Larimer FW Hauser LJ Prodigal Prokaryotic Dynamic Programming Genefinding Algorithm. BMC Bioinformatics 2010; 11**:** 119 10.1186/1471-2105-11-119 20211023 PMC2848648

[19] Pati A Ivanova N Mikhailova N Ovchinikova G Hooper SD Lykidis A Kyrpides NC GenePRIMP: A Gene Prediction Improvement Pipeline for microbial genomes. Nat Methods (*in press*) 10.1038/nmeth.1457 20436475

[20] Markowitz VM Mavromatis K Ivanova NN Chen IMA Chu K Kyrpides NC IMG ER: a system for microbial genome annotation expert review and curation. Bioinformatics 2009; 25:2271-2278 10.1093/bioinformatics/btp393 19561336

